# Future implications of artificial intelligence in lung cancer screening: a systematic review

**DOI:** 10.1093/bjro/tzae035

**Published:** 2024-10-15

**Authors:** Joseph Quirk, Conor Mac Donnchadha, Jonathan Vaantaja, Cameron Mitchell, Nicolas Marchi, Jasmine AlSaleh, Bryan Dalton

**Affiliations:** Trinity College Dublin School of Medicine, Trinity Biomedical Sciences Institute, 152-160 Pearse Street, Dublin 2, D02R590, Ireland; St James’s Hospital, James’s Street, Dublin 8, Dublin, D08 NHY1, Ireland; Trinity College Dublin School of Medicine, Trinity Biomedical Sciences Institute, 152-160 Pearse Street, Dublin 2, D02R590, Ireland; Tallaght University Hospital, Tallaght, Dublin 24, Dublin, D24 NR0A, Ireland; Trinity College Dublin School of Medicine, Trinity Biomedical Sciences Institute, 152-160 Pearse Street, Dublin 2, D02R590, Ireland; Aintree University Hospital (Liverpool University Hospitals Foundation Trust NHS), Lower Ln, Fazakerley, Liverpool, L9 7AL, United Kingdom; Trinity College Dublin School of Medicine, Trinity Biomedical Sciences Institute, 152-160 Pearse Street, Dublin 2, D02R590, Ireland; Vassar Brothers Medical Center, Nuvance Health, 45 Reade Pl, Poughkeepsie, NY 12601, United States; Trinity College Dublin School of Medicine, Trinity Biomedical Sciences Institute, 152-160 Pearse Street, Dublin 2, D02R590, Ireland; University Hospitals Plymouth NHS Trust, Derriford Road, Plymouth, Devon, PL6 8DH, United Kingdom; Trinity College Dublin School of Medicine, Trinity Biomedical Sciences Institute, 152-160 Pearse Street, Dublin 2, D02R590, Ireland; Mubarak Al Kabeer Hospital, Street 103, Jabriya, Kuwait; Radiology Department, St James’s Hospital, James’s Street, Dublin 8, Dublin, D08 NHY1, Ireland

**Keywords:** artificial intelligence, radiology, lung cancer, screening

## Abstract

**Objectives:**

The aim of this study was to systematically review the literature to assess the application of AI-based interventions in lung cancer screening, and its future implications.

**Methods:**

Relevant published literature was screened using PRISMA guidelines across three databases: PubMed, Scopus, and Web of Science. Search terms for article selection included “artificial intelligence,” “radiology,” “lung cancer,” “screening,” and “diagnostic.” Included studies evaluated the use of AI in lung cancer screening and diagnosis.

**Results:**

Twelve studies met the inclusion criteria. All studies concerned the role of AI in lung cancer screening and diagnosis. The AIs demonstrated promising ability across four domains: (1) detection, (2) characterization and differentiation, (3) augmentation of the work of human radiologists, (4) AI implementation of the LUNG-RADS framework and its ability to augment this framework. All studies reported positive results, demonstrating in some cases AI’s ability to perform these tasks to a level close to that of human radiologists.

**Conclusions:**

The AI systems included in this review were found to be effective screening tools for lung cancer. These findings hold important implications for the future use of AI in lung cancer screening programmes as they may see use as an adjunctive tool for lung cancer screening that would aid in making early and accurate diagnosis.

**Advances in knowledge:**

AI-based systems appear to be powerful tools that can assist radiologists with lung cancer screening and diagnosis.

## Introduction

Lung cancer is the leading cause of cancer-related mortality worldwide. In 2020, it was estimated that 2.2 million cases of lung cancer were newly diagnosed and approximately 1.8 million people died from the disease.^1^ It is the leading cause of cancer death in men and the third leading cause of death in women, accounting for 21.5% and 13.7% of all cancer deaths, respectively.^1^ Despite advancements in the molecular biology of lung cancer and the emergence of novel nanomedicine technology for its treatment and management, mortality remains high.^2^^,^^3^ Nearly 50% of patients with lung cancer are diagnosed at an advanced stage of cancer progression and less than 10% of patients are clinically asymptomatic at the time of diagnosis.^2^^,^^4^ This review was conducted in Ireland, where most lung cancers are diagnosed at stages III and IV.^5^ This can be attributed to a lack of lung cancer screening programmes in Ireland. Additionally, lung cancer incidence is expected to rise by up to 136% in females and by up to 52% in males by 2040.^6^ Low-dose CT screening for at-risk patients has been identified as a potential means of detecting lung cancer at an earlier stage.^5^ Furthermore, no classification guidelines exist to distinguish cancerous pulmonary nodules from benign ones upon first identification during lung cancer screening.^2^^,^^4^ This can potentially lead to overdiagnosis and the unnecessary treatment of clinically benign findings.^7^

The desire to improve the efficacy of lung cancer screening processes has led to innovations in the field of clinical radiology, in particular, the use of artificial intelligence (AI) and medical imaging. The objective of this systematic review was to assess the application of AI-based interventions in lung cancer screening and detection, and what future implications it may have in lung cancer screening.

## Study design and methods

Articles in this review were retrieved from three databases: PubMed, Scopus, and Web of Science. The Patients/Intervention/Comparison/Outcome (PICO) model was used to develop a search strategy to identify suitable papers (see Table 1). The Preferred Reporting Items for Systematic Reviews and Meta-Analyses (PRISMA) guidelines outlined the systematic selection process of our study.^8^^,^^9^

**Table 1.** PICO model.

PICO criteria	Determinants
Patient, population, or problem	Screening phase of lung cancer
Intervention	Radiology aided by artificial intelligence
Comparison	Clinical based radiology
Outcome	Accuracy—true positives, true negatives
Study	Clinical

**Figure 1.**
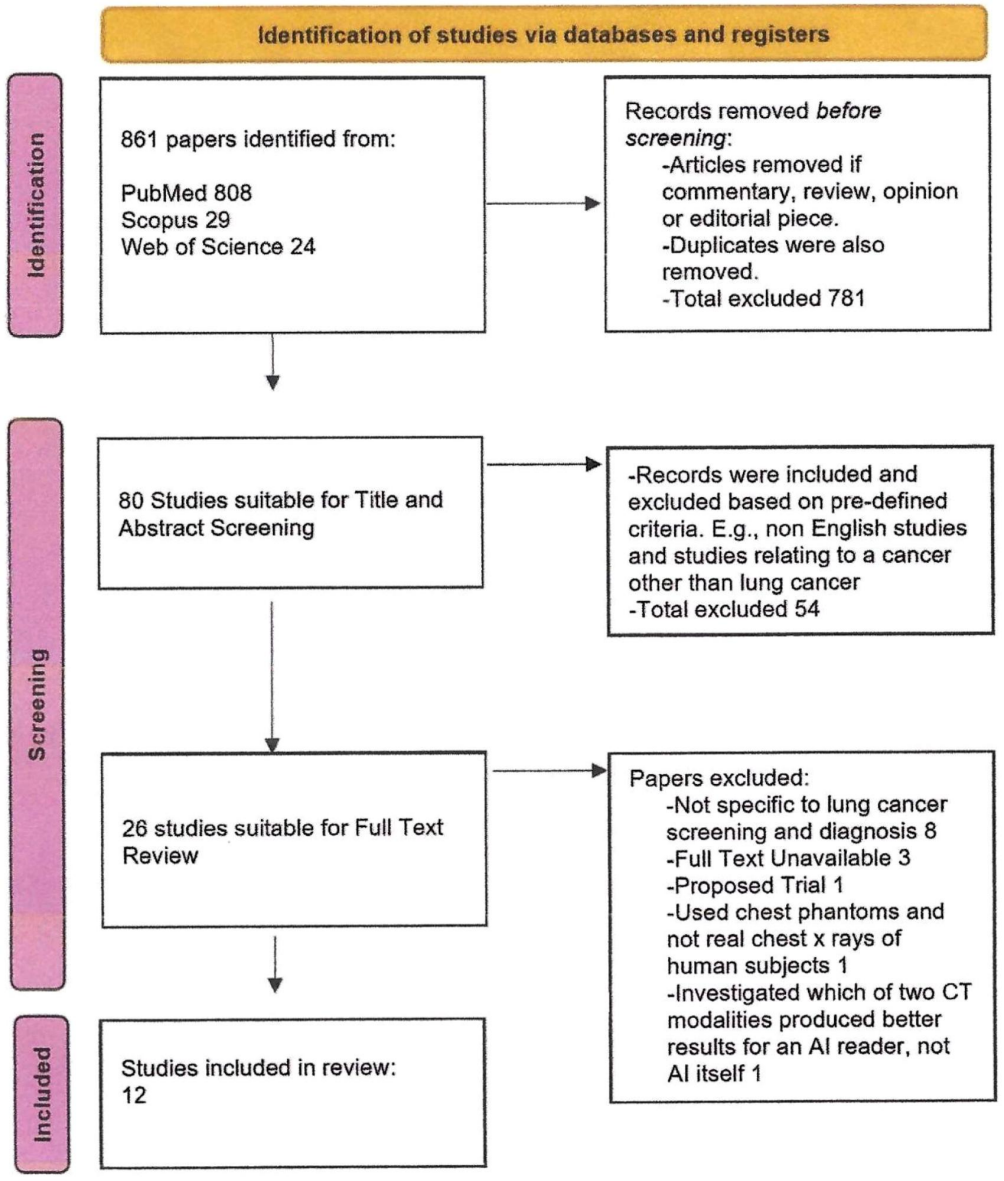
PRISMA identification of studies via databases and registers.

Search terms for article selection included the keywords “artificial intelligence” AND “radiology” AND “lung cancer” AND “screening” AND “diagnostic.” The range of publication dates included in our search were those articles published between January 2000 and October 2023. This search was conducted between October 27 and 31, 2023 and an overview of our systematic search results can be found in the PRISMA flow diagram (see Figure 1).

### Inclusion and exclusion criteria

Papers were included if they met the following inclusion criteria: patients were referred to radiology for lung cancer diagnosis, and researchers studied an AI intervention in the screening/diagnostic phase of lung cancer identification. In the context of this review, AI was defined as the use of machine learning (ML) algorithms, such as deep learning (DL) and its iterations including convolutional neural networks (CNN) and computer-aided diagnosis (CAD). Machine learning refers to algorithms that aim to emulate human intelligence by learning from data inputs and outputs to recognise patterns without being directly programmed.^10^ DL is a subfield of AI and ML, upon which most new AI innovations are based, which employs multiple layers of computation to scrutinise data with multiple layers of abstraction, such as images (to which CNNs are particularly suited).^11^ A CNN is a DL modality well suited to analysing visual data, characterised by a multi-layered model that receives inputs and builds on outputs of other layers within the model to recognise patterns; CNNs are often used in image recognition tasks.^12^

Only papers written in the English language were included. Studies were excluded if they considered conditions other than lung cancer. Studies were excluded if they did not fulfil our definition of AI or if the study was not in the context of the screening/diagnostic phase of lung cancer. The inclusion and exclusion criteria are summarised in table format (see Table 2).

**Table 2.** Inclusion and exclusion criteria for paper selection.

Inclusion	Exclusion
Clinical studies on human subjects, including the use of scan datasets	Opinion pieces, commentary texts, and systematic reviews
Studies included if part of the screening/diagnostic phase of lung cancer (including looking at the identification and quantification of tumour)	Study excluded if looking at other conditions or for general use
An AI intervention was utilised	Studies excluded if no AI intervention was utilised
Study available in English language	Non-English language studies
	Animal studies

### Selection of reviewed papers

Following the selection of all clinical studies, two authors independently graded the titles for relevance—marking “1” for relevant and “0” for irrelevant. Any disagreement was resolved by discussion and consensus with a third author. At this time, all remaining duplicates were removed from relevant studies. The same process was repeated for abstract screening.

## Results

### Characteristics of studies and populations

Eight hundred sixty-one papers were identified from three scientific online libraries—PubMed, Scopus, and Web of Science. The exclusion of contemporary pieces, editorials, reviews, and duplicates yielded 80 papers whose titles and abstracts were then evaluated for relevance. Fifty-four studies were excluded at this stage for being irrelevant and for not meeting our inclusion/exclusion criteria described previously. This left 26 studies for full-text review. Upon full-text review, eight studies were removed as they were not specific to lung cancer screening and diagnosis. Three studies were removed because the full text was unavailable. One study was removed as it was a proposed trial and not a completed trial. One study was removed because it used a non-human population (chest phantom radiographs). Another study was removed as it investigated which CT modality was superior for AI, not the ability of an AI itself. This process resulted in the inclusion of 12 studies in the systematic review, as displayed by the PRISMA flowchart (see Figure 1).

All studies included in this review concerned AI algorithms being applied to the screening/diagnostic phase of lung cancer *via* clinical CT scans or chest X-rays and/or CT datasets (see Table 3).

**Table 3.** Characteristics of the studies included in the review.

Author (year)	Study type	Objective of study	Sample size	Radiology conditions	Algorithm conditions	Outcome variables	Main finding(s)
Adams et al (2023)	Retrospective	Evaluation of new AI-based image classifier	963 patients	CT Scan	CAD-RevealAILung, commercial algorithm	The AI classifier’s AUC was compared to existing tools. Measurement of number of false positive findings and unnecessary investigations when compared to Lung-RADS alone	The mSI generated by the AI performs as well as existing clinical risk models (AUC = 0.89 vs 0.86-0.88). Provides more accurate detection of malignancy risk when combined with existing models, eg, Lung-RADS
Armato et al. (2005)	Retrospective	Evaluation of measurements	22 mesothelioma patients	CT scan	Three algorithms used Local max-distance algorithm Normal to initial endpoint algorithm Hybrid algorithm Algorithms were a research effort	Observer acceptance rate, measurement acceptance with modification <2 mm	Hybrid highest acceptance rate 86% and 95% measurements changed by <2 mm
Chamberlin et al (2021)	Retrospective	Performance of detection	117 patients (no diagnosis)	Low-dose CT scan	Two CNNs, AI RAD companion, Siemens Healthineers, commercial algorithm	Interobserver concordance, sensitivity, specificity, prediction of lung cancer outcomes 1-year follow-up	Interobserver concordance CACV 0.9 and LN 0.85; CACV sensitivity = 0.929 & specificity = 0.960; lung nodules sensitivity = 1 & specificity = 0.708; 1-year follow up = AUC lung cancer = 0.942
Chauvie et al (2020)	Prospective	Performance of detection	Part of SOS clinical trial—1594 patients	DTS	1—Logistic regression 2—Random forest 3—Neural network Algorithms were a research effort	Positive predictive value and sensitivity	Best predictor = neural network with PPV = 0.95 and sensitivity = 0.90
Cheng et al (2022)	Retrospective	Performance of detection	357 patients with either focal pneumonia or peripheral lung cancer with solitary solid nodule	CT scan	3D CNN Algorithm was a research effort	Both accuracy and AUC were measured in comparison to junior, intermediate and senior doctors when differentiating between focal pneumonia and peripheral lung cancer	Accuracy: 91.596% (CNN) vs 90.48% (junior physician) AUC: 0.946 (CNN) vs 0.957 (junior physician) Both the intermediate and senior physician outperformed the CNN
Nam et al (2022)	Retrospective	Performance of detection	118 patients with Lung-RADS4 classification nodule on CT with both nodule positive and nodule negative chest X rays. 51 without lung nodules on CT or chest X-ray Total = 169	Chest X-ray	CNN-LUNIT INSIGHT CXR 2.0, commercial algorithm	Specificity and sensitivity of radiologists when evaluating category 4 lung nodules on chest X-rays with and without the AI algorithm	Sensitivity of radiologists improved with the algorithm (38.8%-45%) while specificity did not change as much (94.1%-92.2%)
Nguyen et al (2020)	Retrospective	Performance of detection	1—Kaggle 2017 (*n* = 1375) 2—NLST (*n* = 4075 3—LIDC (*n* = 1018) Total = 6468	CT scan	ASEM training of CNN Algorithm was a research effort	Accuracy of lung nodule identification and detection of cancer cases & AUC (sensitivity)	Accuracy of 92% (Kaggle17), 93% (NLST), and 73% (LIDC); Sensitivity of 0.94 (Kaggle), 0.88 (NLST), and 0.81 (LIDC)
Park et al (2021)	Retrospective	Lung-RADS categorization	Randomly selected datasets from NLST (*n* = 200)	CT scan	CAD- VUNO MEDLUNG CT AI v1.01, commercial algorithm	CAD impact on inter-reader agreement—with CAD and without CAD	With CAD readers upstaged Inter-reader agreement improved slightly with CAD Disagreement of nodule size reduced from 5.1% to 3.1%
Shah et al. (2005)	Retrospective	Evaluation of ability to differentiate nodules	35 patients of known diagnoses (19 M/16 B)	CT scan	CAD 1—feature selection step 2—Classifier 1 3—Classifier 2 4—Classifier 3 Algorithms were a research effort	AUC (sensitivity)	Sensitivity range 0.69-0.92—classifier based on logistic regression performed the best with 0.92
Yoo et al (2021)	Retrospective	Evaluation of ability to improve performance	96 cancer positive patients, 196 cancer negative patients Total = 292	Chest X-ray	CNN LUNIT INSIGHT for Chest Radiography, v2.4.11, commercial algorithm	Measured sensitivity and false positive rate of residents and radiologists with and without AI	Average sensitivity of residents increased when using AI (0.61-0.72) whereas radiologists’ sensitivity did not change as much (0.76-0.76). False positives were similar for residents using AI but decreased in radiologists using AI
Zhang et al (2022) Lung Nodule Detectability	Retrospective	Compare lung nodule detection in actual radiology reports and AI-assisted reading	860 asymptomatic patients aged 45-74 screened between Nov and Dec 2019 (retrospective)	CT scan (low-dose, non-contrast)	InferRead CT lung—consists of two R-CNN models merged into one programme Algorithms were a research effort	Diagnostic sensitivity and specificity of radiologist observation vs AI-assisted analysis of CT thorax for solid and non-solid lung nodules	AI sensitivity 0.988-1 for solid and non-solid nodules; radiologist 0.252 for non-solid and 0.524 for solid nodules, respectively
Zhang et al (2022) Radiomics	Retrospective	A study to determine diagnostic and prognostic value of deep learning/radiomics for solid lung nodules	720 patients with 720 nodules - 348 benign and 372 malignant (diagnosed by surgery or biopsy)	CT scan	3D CNN and Random Forest (RF) models Algorithms were a research effort	Diagnostic performances of CNN w/clinical features and RF models w/radiomics features and radiologists in solid lung nodules; AUC/sensitivity	CNN w/clinical features sensitivity 0.778, RF w/radiomics features sensitivity 0.747, junior radiologist sensitivity 0.884; models had higher specificity than radiologists (around 0.5 vs 0.6-0.7)

Abbreviations: ASEM = active semi-supervised expectation maximization; AUC = area under curve ∼ sensitivity; CAD = computer-aided diagnosis; CNN = convolutional neural network; DTS = digital tomosynthesis; LIDC = Lung Image Database Consortium; NLST = National Lung Screening Trial; SOS = Studio OSservazionale.

### Characteristics and outcomes of studies

All studies concerned the role of AI in lung cancer screening and diagnosis. The characteristics and details of the studies are outlined in Table 3. All studies investigated chest radiographs or CT scans at a single point in time. All studies except for one were retrospective studies. Five studies evaluated AI’s performance in terms of lung nodule detection.^13–17^ One study graded AI’s ability to measure cancerous lesions.^18^ Another study tested an AI’s ability to score nodules based on the Lung-RADS scale^19^ and a further study measured an AI’s capacity to differentiate lung nodules.^20^ Another study investigated an AI-based image classifier and compared it to Lung-RADS.^21^ Three studies investigated an AI’s ability to augment and improve the diagnoses of radiologists.^22–24^ The range of subjects involved in each study was 22 to 6468, resulting in data collection from a total of 11 797 subjects. Three studies used the CAD algorithm.^19–21^ Six studies used a CNN.^13^^,^^15^^,^^16^^,^^22–24^ Three studies used multiple algorithm models, such as radiomics.^14^^,^^17^^,^^18^ Radiomics is an evolving field which involves the extraction of quantitative features from medical images, permitting more rigorous analysis and pattern recognition^25^. One study tasked AI with reading digital tomosynthesis (DTS) X-rays.^14^ Two studies used chest X-rays.^22^^,^^23^ All other studies used CT images.

### Main findings

The studies investigated the ability of AI to differentiate, measure, count, and grade lung nodules or other cancerous lung lesions. Most studies showed positive results, demonstrating AI’s ability to achieve these tasks and produce readings that were close to that of radiologists.

As previously described, five studies evaluated lung nodule detection. Sensitivity rates were 1.00,^13^ 0.90,^14^ 0.946,^15^ 0.94,^16^ and 0.778,^17^ respectively. The two studies which tested three different AI approaches found the neural network^14^ and the hybrid approach^18^ to be most efficacious. The hybrid approach had an 86% acceptance rate. The CAD used by Park et al demonstrated AI’s potential to accompany radiology teams as it increased inter-reader agreement and decreased nodule size disagreement by 2%.^19^ Shah et al also used a CAD which produced a range of sensitivity values (0.69-0.92) for nodule differentiation depending on which AI modality was selected.^20^ The three studies which investigated AI’s ability to improve the accuracy of radiologists’ diagnoses each showed positive results.^22–24^ Two of these studies showed that the radiologists’ sensitivity improved when using AI-assisted reading,^22^^,^^24^ whereas one study showed that only residents’ sensitivity increased while radiologists’ sensitivity remained unchanged with AI.^23^ Finally, Adams et al used AI to classify CT findings and developed a new risk index called malignancy similarity index (mSI).^21^

### Detection of lung cancer

Studies conducted by Chamberlin et al,^13^ Chauvie et al,^14^ Cheng et al,^15^ Nguyen et al,^16^ and Zhang et al^17^ all look at quantifying the performance of AI algorithms in detecting cancerous areas in the lung. Nguyen et al^16^ demonstrated active semi-supervised expectation maximum training for CNN for lung cancer screening using CT can generate performance numbers comparable to that of supervised CNN training but with more than half the training data set. Semi-supervised learning refers to a training method whereby an algorithm is trained on partially labelled datasets. The “active learning” refers to the help provided by a human “oracle” who labels some of the unlabelled data for the algorithm.^16^ One advantage of semi-supervised learning is that it allows training to be performed on a wider array of data sets as fully labelled datasets are much less common than unlabelled ones. Chamberlin et al^13^ showed conclusive evidence that across 117 patients who underwent a low-dose CT scan, the agreement of AI findings with experts was excellent (0.846-0.904) with high sensitivity (0.929-1) and specificity (0.708-0.960). The result of these findings leads to a significant improvement of the prediction of major cardiopulmonary outcomes at 1-year follow-up, specifically lung cancer by 0.942. Chauvie et al^14^ provided quantitative value as to why AI screening has an important place in lung cancer screening. Results showed that the implementation of AI algorithms, specifically neural networks, to the lung cancer screening phase enhanced the positive predictive value (PPV) of chest DTS, and the potential to help radiologists reduce false positives and false negatives. Cheng et al^15^ investigated an AI’s ability to detect lung nodules versus three sets of radiologists: junior physicians (3 years subspeciality experience), intermediate physicians (5 years experience), and senior physicians (10 years experience). The CNN used had a detection rate of 91.596% and an area under the curve (AUC) of 0.946, similar to that of a junior physician (90.48%, 0.957). However, both the intermediate (94.96%, 0.989) and senior physician (96.92%, 0.980) outperformed the CNN. This study demonstrates AI’s ability to detect lung nodules without the help of physicians. Still, it was only able to perform at the level of a junior physician, suggesting that AI may help as a screening tool but still requires senior supervision and input. Zhang et al explored the performance of two AIs, a CNN and a radiomics model, when detecting solid pulmonary nodules.^17^ Their performance was compared to junior radiologists. The CNN based model had the highest specificity (0.778), followed by the radiomics based model (0.606) which in turn outperformed the junior radiologists (0.486). However, the junior radiologists had the highest sensitivity (0.884), followed by the CNN based model (0.778) which in turn outperformed the radiomics based model (0.747). These results again suggest that current AI models are able to detect lung nodules with a similar accuracy to that of junior radiologists.

## Discussion

There are multiple areas from our review which describe how AI integration may assist in clinical radiology and lung cancer screening. Four main themes emerged and these may have important implications for the future use of AI in lung cancer screening programmes: (1) its increased ability to detect nodules without human assistance, (2) its ability to acquire accurate measurements of suspicious nodules as well as characterise them and differentiate malignant from benign lesions, (3) its ability to augment the work of human radiologists, (4) its ability to automatically categorise findings via Lung-RADS and to augment the Lung-RADS tool with a new scoring system.

### Characterization of lesions

#### Lesion segmentation

Armato et al^18^ seemingly built off the work of Shah et al^20^ and evaluated the ability of three AI algorithms to measure tumour size, a fundamental task in the screening and diagnosis of lung cancer. One algorithm of note, which was a hybrid model, had an interobserver acceptance rate of 86% and a measurement acceptance rate of 95% with a < mm change in dimension. These results suggest that AI algorithms have the ability to accurately first detect lung cancer and then calculate tumour size comparable to that of clinical radiologists. Thus making the case AI has the ability to localise cancerous regions in the lung and provide accurate measurements. This demonstrates that an automated system can make screening more efficient and less time-consuming than the manual acquisition of measurements.

#### Differentiation between malignant and benign findings

Shah et al^20^ evaluated the decision-making process of AI in differentiating known diagnoses of nodules which were confirmed by a thoracic radiologist. The results showed CAD (an early form of AI) had the ability to make accurate decisions when differentiating malignant vs benign, with a sensitivity score ranging from 0.69 to 0.92 across three algorithms. The third, a logistic regression, scored the best of the CAD algorithms used. The sensitivity also increased when observer input occurred with CAD, reaching 0.95 in most cases.^20^ Thus highlighting early evidence that a CAD had the capability and potential to one day be adopted in a combined approach of screening for lung malignancy.

### AI-assisted reading of chest imaging

Yoo et al employed a CNN model to assist physician reading of chest radiographs (CXR) four weeks after they had initially reviewed them without any aid.^23^ They found that with AI, sensitivity of junior radiologists in detecting lung cancers increased by 11%; specificity remained the same. While senior radiologists' sensitivity did not increase significantly with AI, their specificity did, by 7%, and they had fewer false positives. It was shown, therefore, that AI has benefits in different regards depending on seniority, with specificity improving among experienced radiologists and trainees improving in sensitivity; all while generating fewer false positives.

Similarly, Nam et al used a CNN model to help radiologists on a second reading of CXRs looking for lung nodules.^22^ In their study, they showed that radiologists aided by the algorithm increased their sensitivity for lung nodules by 6.3% without a change in specificity or false positive rates. While this is a more modest effect, the authors underline that only single PA CXRs were analysed, without referring to previous scans or lateral imaging.

Zhang et al demonstrated the most emphatic improvement in nodule detection with AI-assisted reading.^24^ Unlike the previous two studies, the authors examined AI-assistance in reading of CT scans. Radiologist sensitivity increased by 46.4% for solid nodules and by 73.9% for non-solid nodules after the algorithm helped the physician. Again, AI-assisted reading of the scans did not result in reduced specificity.

### AI and Lung-RADS framework

Park et al^19^ looked at the ability of an AI algorithm to automatically classify CT findings *via* Lung-RADS—a standardised triage system to classify radiological findings. One significant limitation of the validated Lung-RADS framework for the management of lung nodules on CT is its observer interobserver variability, as radiologists manually place cursors during node measurement which can lead to measurement bias.^26^ Park et al assessed the AI’s performance then matched to radiological observers. This novel study on AI’s decision-making process did not find significant improvements in the agreement of Lung-RADS categorization, however, it did demonstrate an ability to reduce measurement variability and discrepancies between radiologists.

Adams et al tested a machine learning model called RevealAI-Lung to classify malignancy risk of lung nodules on CT imaging.^21^ They hypothesised that its implementation, along with Lung-RADs, would enhance the management of these CT findings in both screening and all-cause scans. Using data from the NSLT cohort to train the model, they developed a malignancy risk score called mSI. Of note, the National Lung Screening Trial (NLST) defined a suspicious nodule as positive if its diameter was ≥4 mm and if it was not calcified. They demonstrated that adding mSI to Lung-RADS significantly enhanced sensitivity and specificity and reduced false positive rate if nodule characteristic data was reported. For example, mSI plus Lung-RADS increased sensitivity by 68% (*P* < .001) and specificity by 17% (*P* < .001) within the NLST, compared to Lung-RADS alone. The researchers also demonstrated that the addition of mSI with Lung-RADS, decreased diagnostic delay of subsequent scans of high-risk nodules by 33%-45%.^23^ Overall, this study demonstrated the potential clinical utility of using a novel index score developed through AI machine learning, in conjunction with the existing Lung-RADS framework. The promising results may lead to a decrease in inappropriate, often invasive follow-up investigations for lung nodules on CT imaging and lead to earlier detection of high-risk malignant findings.

### Limitations of our study

A limitation of our review is the search strategy employed. It is possible that some citations were omitted secondary to only including studies published in English. Additionally, human error in the screening process may have impeded the number of studies found, leading to false negatives (inter-reviewer error). However, our adoption of blinded title and abstract screening, with a third reviewer to go over any disagreements, was used to reduce false negatives and positives. Finally, it was noted that most studies incorporated into this review were retrospective. This allows the studies to be compared more readily due to their similar nature. However, the preponderance of retrospective studies in this review means that if there were a bias towards AI in such studies, it would be difficult to detect, as there is less comparison available in the form of prospective study data.

### Future implications of AI in healthcare

AI technology has the potential to improve health outcomes for patients as well as increase efficiencies in the healthcare system. Additionally, by introducing lower-cost care provided by AI, a greater degree of health equity can be achieved. Work by Guo and Li found that the introduction of AI technology in rural areas of developing nations was able to compensate for the lack of physician access and improve the availability of health care services.^27^ From a macroeconomic perspective, AI in the health industry is projected to have far-reaching effects. By 2030, AI is predicted to add $15.7 trillion to the global economy. The health sector has been identified as the industry most likely to experience disruption due to AI innovation.^28^ In Europe specifically, AI could save health systems between €170.9 and €212.4 billion annually.^29^ AI has also recently been shown to be a cost effective strategy in low-dose CT screening for lung cancer.^30^ From a system-level perspective, AI technology offers capabilities to improve and expand offerings to patients and positively benefit the economy as a whole.

## Conclusion

### Conclusion and implications of AI in future lung cancer screening programmes

In light of there being no definitive lung cancer screening initiative in countries such as Ireland, many diagnoses are not made until the advanced stages of the disease process.^5^ As of now, low-dose CT screening for at-risk patients has been identified as a potential means of detecting lung cancer at an earlier stage.^5^ If such policy changes were to be made to implement lung cancer screens, AI should be considered to lessen the burden on radiologists. As highlighted, AI application to lung cancer screening of radiological scans has proven highly effective in differentiating malignant vs benign tumours, acquiring accurate measurements of said tumours, detecting lung cancer at a level of highly trained radiologists, and accurately categorising findings. Moreover, the efficacy of AI may be underestimated as most studies in this review use only one type of radiological scan, without reference to other modalities or previous investigations. All of these findings have major implications on not only making an early and accurate diagnosis but initiating effective therapeutic approaches thereby improving patient outcomes. While it is unlikely that in the next 15 years AI will replace the role of radiologists, AI is likely to become an essential adjunctive tool in the clinical decision-making process in clinical radiology.

## Review protocol

No review protocol has been registered.
